# Neural Pathway of Renovative and Innovative Products Appreciation

**DOI:** 10.1038/srep38800

**Published:** 2016-12-12

**Authors:** Furong Huang, Chiyue Chiu, Jing Luo

**Affiliations:** 1 Beijing Key Laboratory of Learning and Cognition, The Collaborative Innovation Center for Capital Education Development, Department of Psychology, Capital Normal University, Beijing, China; 2 College of Psychology, Jiangxi Normal University, Nanchang, China; 3 Faculty of Social Science, The Chinese University of Hong Kong, Hong Kong Special Administrative Region; 4 Key Laboratory of Mental Health, Institute of Psychology, Chinese Academy of Sciences, Beijing, China

## Abstract

According to the level of change an invention makes on existing things and how it overrides people’s mental schemas on established categories, new inventions can be classified into two groups: incremental inventions (i.e., renovations), which make minor improvements on existing designs, and radical inventions (i.e., innovations), which make major developments that enable people to do things they have never been able to do before. Although innovation and renovation are two fundamentally different types of creation that feature new changes ranging from those in product development to those in large scale social changes, and people tend to report higher subjective preferences for incremental inventions compared to radical inventions, the cognitive brain mechanisms underlying the mental representation of these two types of inventions remains unknown. Through the use of innovative and renovative designs as materials, we found that relative to non-creative designs, creative (renovative &innovative) designs enhanced memory or association-related activation in the right parahippocampus. In particular, innovations evoked more activation in the conceptual pathway for representing objects than did renovations, whereas renovations evoked more activation in the motor pathway than innovations. These results suggest that operating experiences may provide advantages for understanding and appreciating creative designs.

Although invention can occur in countless ways, it is generally categorized as either an incremental or radical invention based on the level of change compared to existing things or products^1^,^2^,^3^. Incremental inventions involve direct improvements and modifications to existing things or products, such as minor changes in technology, through which the system’s efficiency or product’s performance is improved. In contrast, radical inventions are the result of a major technological or methodlogical breakthrough that enables people to do what they have never been able to do before^4^,^5^. This “incremental vs. radical” feature of change characterizes all types of inventions in all fields. For example, the arguments of “evolution vs. revolution” in large scale social change differ regarding people’s preference for a gradual and continuous development of fundamental social morals and ideas or the preference for radical and sudden social reforms and changes^6^. Likewise, “renovation vs. innovation” in the business field and product development differs in the selection of minor changes to improve current approaches or technologies while leaving their essence intact compared to the adoption of truly new approaches and the abandonment of old ones. Such as Intel was transformed from a semiconductor memory company into a semiconductor chip-making company^7^.

In spite of the fundamentality and essentiality of the difference between incremental and radical inventions, the cognitive brain mechanisms through which these two types of inventions are mentally represented remain unclear. The aim of this study was to investigate the neural correlates of the incremental and radical types of inventions by using new creative product designs as examples. Although the representational difference between the incrementally and radically creative product designs may have domain-specific limitations and cannot be applied to all creative fields, it can reflect the essential difference between renovation and innovation in a well-defined and strictly controlled experimental situation.

Previous studies on people’s understanding and accepting of creative new products or designs have revealed that how the positive and negative aspects of the newness attributes of a new product are evaluated may depend on the invention level^8^,^9^,^10^,^11^,^12^,^13^. The positive effects of novel attributes hold only in the case of incremental inventions, whereas for radical inventions, the newness attributes can actually reduce product evaluations^8^,^9^. In particular, consumers may interpret the newness attributes of incremental inventions as additional benefits, whereas these attributes of radical inventions may lead consumers to feel they lack the ability to comprehend product features or make effective use of them, resulting in interferences of increased learning costs^9^,^10^ and great uncertainty regarding benefits and risks^11^,^12^,^13^. Thus, people tend to report higher subjective preferences for incremental inventions compared to radical inventions^14^,^15^. With respect to these above-mentioned complex individual feelings and attitudes, however, the fundamental cognitive processes and neural activities that underlie the appreciation of incremental and radical creative products remain largely unclear. Revealing how individuals understand and appreciate these two fundamentally different types of creative ideas or products has important commercial, educational, and sociocultural implications.

To understand the usage and benefits of new products, individuals must transfer previous knowledge and experience through analogical learning and mental simulation^16^. Or rather, individuals can construct a mental representation of creative products by using information already contained in familiar product categories^17^,^18^. Firstly, we assume that both the mental representation of incrementally and radically creative products will be construct by the medial temporal lobe (MTL) structure, which has been demonstrated to be critical for memory encoding and storage of new things^19^,^20^, and particularly associated with the perception of novel objects^21^. Secondly, we propose that the key difference between the mental representation of incrementally and radically creative products is related to manipulating skills and conceptual knowledge factors. Particularly, incrementally creative products may be understood more depend on one’s operational experiences of the existing prototype, whereas radically creative products may require more extensive semantic and conceptual analyses of its function because little practical experience can be retrieved directly. In the field of human tool use, two systems have been demonstrated to subserve manipulable object processing: the first is conceptual pathway, which primarily includes the inferior frontal gyrus (IFG) (BA44, 45) and posterior middle temporal lobe (pMTG) to extract conceptual knowledge concerning tools and their functions (what a product is for); the second system is motor pathway, specifically, includes the posterior parietal cortex (PPC) and the premotor cortex (PMC), which are responsible for the motor skills necessary for performing actions (how to use a product)^22^,^23^,^24^. Therefore, the hypothesis was that the motor and conceptual pathways are more important in the processing of incrementally and radically creative products, respectively.

## Results

### Behavioral Results

Following online and offline screening, there were, on average, 31, 26, and 26 valid trials for the ordinary, renovative, and innovative conditions, respectively. The following analyses were performed only on the valid trials in each condition.

For the mean online reaction times, the difference among the three conditions was significant [*F*(2,38) = 64.48, *p* = 0.000, η_p_^2^ = 0.77]. Post hoc contrasts indicated that the reaction times for both the renovative (2379±345 ms) and innovative designs (2484±405 ms) were higher than those for the ordinary designs (1923±315 ms) [*p* = 0.000]; however, there was no difference between the renovative and innovative designs [*p* = 0.123].

### Whole-Brain fMRI Results

**Figure 1:**
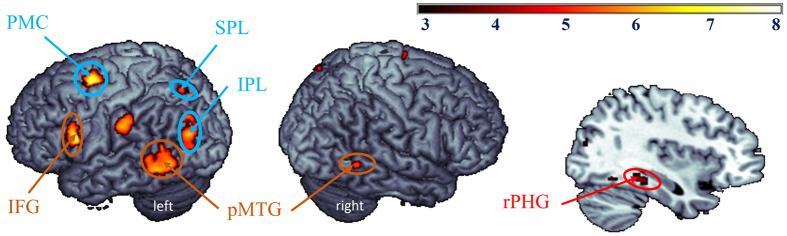
Brain activations in the “renovatibe & innovative minus ordinary designs” contrast. IFG, inferior frontal gyrus; IPL, inferior parietal lobule; PMC, premotor cortex; pMTG, posterior middle temporal gyrus; rPHG, right parahippocampal gyrus; SPL, superior parietal lobule.

To identify the brain regions activated in the processing of creative designs, i.e., the renovative and innovative designs, compared with the non-creative designs, i.e., the ordinary designs, we performed the “renovation & innovation > ordinary” contrast. Brain activations were identified in the left IFG, PMC, SPL, IPL, somatosensory cortex, right PHG and bilateral pMTG (Table 1 and Fig. 1).

### ROI Results

**Figure 2:**
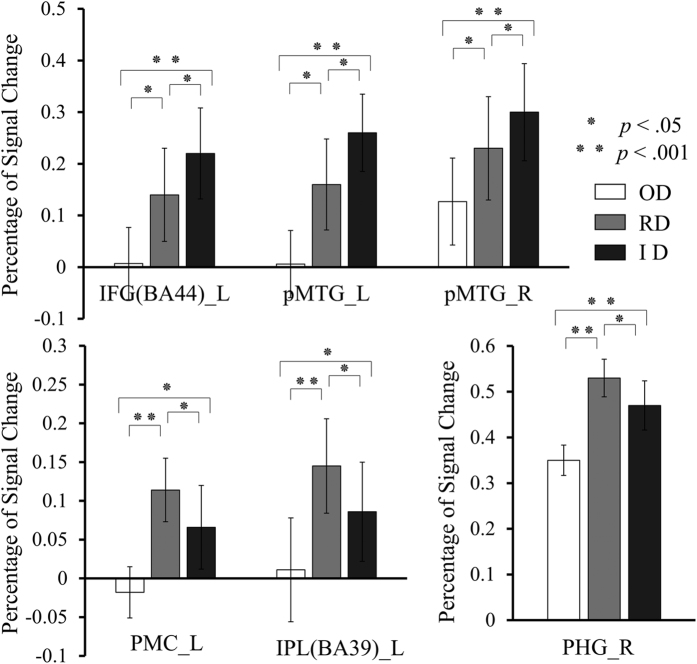
The percent signal changes for ROIs across the three experimental conditions. IFG, inferior frontal gyrus; IPL, inferior parietal lobule; PMC, premotor cortex; PHG, right parahippocampal gyrus; pMTG, posterior middle temporal gyrus. L, left; R, right. OD, ordinary designs; RD, renovative designs; and ID, innovative designs. Error bars represent the 95% confidence intervals. The asterisks indicate significant differences between the conditions (*p < 0.05, **p < 0.001).

For all the left IFG, PMC, SPL, IPL, right PHG and bilateral pMTG ROIs, one way ANOVA demonstrated a significant difference in the percent signal changes among the three conditions (*p*_*s*_ < 0.005). Post-hoc comparisons indicated that the percent signal changes within the left IFG and bilateral pMTG ROIs were significantly increased in the innovative condition compared with both the renovative and ordinary conditions, and these changes were also larger in the renovative condition compared with the ordinary condition (*p*_*s*_ < 0.05, Fig. 2). The percent signal changes within the left IPL, PMC and right PHG ROIs were significantly increased in the renovative condition compared with both the innovative and the ordinary conditions, and these changes were also increased in the innovative condition compared with the ordinary condition (*p*_*s*_ < 0.05, Fig. 2).

### PPI Results

**Figure 3:**
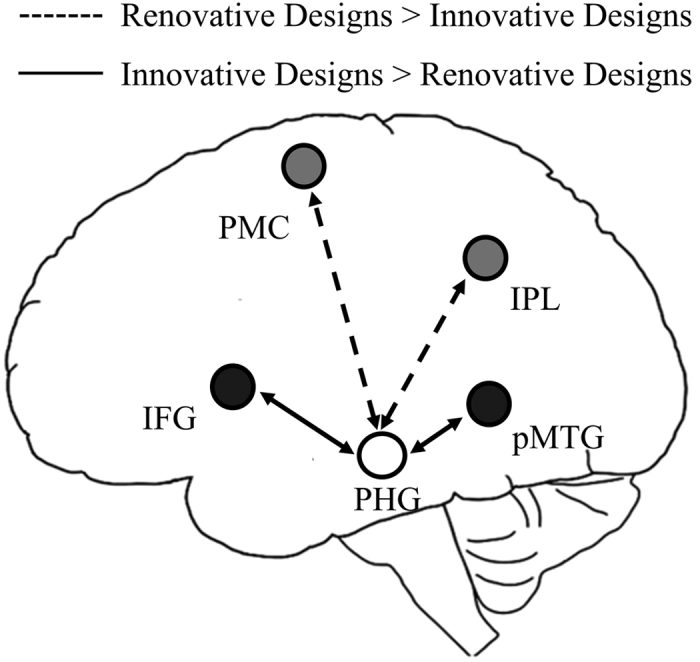
Psychophysiological interaction (PPI) analysis results. Brain areas that exhibit significant PPI from the regions-of-interest in the right PHG when the subjects appreciate the renovative designs compared with the innovative designs, and the innovative designs compared with the renovative designs. IFG, inferior frontal gyrus; IPL, inferior parietal lobule; PHG, parahippocampus; PMC, premotor cortex; pMTG, posterior middle temporal gyrus.

The PPI analysis indicated that during the understanding and evaluation of the renovative designs, the right PHG exhibited stronger functional connectivity with the left PMC and IPL, whereas during the innovative designs, the right PHG exhibited extensive increases in functional connectivity with the left IFG and pMTG (Table 2 and Fig. 3).

## Discussion

Although people tend to report higher subjective preferences for incremental inventions compared to radical inventions, the mental processes and neural mechanisms underlying the evaluation of creative products remain unknown. This fMRI study compared the brain activities of people appreciating creative designs, including both innovative and renovative designs, with the brain activity induced by ordinary designs. The results showed that a) appreciation of creative designs was accompanied by activation in memory-related PHG and the dual pathway for recognizing and representing manipulable objects, and that b) appreciation of innovative and renovative designs engaged distinct neural pathways; in particular, innovation evoked more activation in the conceptual pathway, whereas renovation evoked more activation in the motor pathway.

Brain regions that are generally associated with the appreciation of creative designs included the left IFG, PMC, SPL, IPL, right PHG and bilateral pMTG.

The left IFG, PMC, SPL, IPL and bilateral pMTG are probably responsible for extracting various information about creative designs. In the field of human tool use, there was a distinction between brain systems responsible for semantic knowledge about tool function and associated actions, and the acquired skills necessary for performing these actions^22^,^23^,^24^. The tool use motor network is reported to include the premotor cortex (PMC) and posterior parietal lobe (IPL, SPL)^25^,^26^,^27^, while the semantic network includes parts of the inferior frontal gyrus (IFG), fusiform gyrus (FG), and posterior middle temporal gyrus (pMTG)^28^,^29^,^30^. In this study, participants were asked to view and judge whether tool design was useful or not, which was similar as pantomime task in the neuroimaging studies of tool use. It was for this reason, the activated IFG and pMTG were interpreted as nodes in the conceptual pathway, whereas the activated PMC, SPL, and IPL were interpreted as nodes in the motor pathway, which probably representing semantic knowledge and motor skills about operable creative designs, respectively, although the function of these areas could be indeed complex in other fields.

The PHG is among the medial temporal lobe structures, which subserve the creation, updating, and maintenance of mental representations through the integration of information processed in distributed neocortical regions that are involved in ongoing cognitive processing^31^,^32^. In the present study, the right PHG most likely subserved the construction of mental representations of creative designs by receiving signals from the dual pathways that represent designs in term of semantic knowledge and motor skills. The PHG and dual pathways were more activated by the innovative and renovative designs than by the ordinary ones, probably reflecting a high demanding for the construction of new representations.

Creative designs appreciation activated the dual pathways of manipulable object recognition generally, however, innovative designs were associated with greater activation in IFG and pMTG that belong to the conceptual pathway, whereas renovative designs were associated with greater activation in PMC, SPL and IPL that belong to the motor pathway. Similarly, the PPI results show that the functional connectivity of the right PHG with nodes in the conceptual pathway was stronger in the innovation condition, whereas the connectivity with nodes in the motor pathway was stronger in the renovation condition. These results demonstrated that the mental representation of innovative designs and renovative designs was constructed through different cognitive brain mechanisms. The possible reason lies in the fact that renovative designs differ only slightly from existing designs, thus, the operational skills of the prototype can be migrated by analogy strategies. However, the innovative designs differ substantially from any existing designs and lack directly transferrable practical experience, therefore, the construction of representations relies primarily on the semantic properties of the design’s functions. In short, the mental representation of innovative and renovative designs may differentially depend on semantic knowledge or manipulating skill factors.

The different cognitive brain mechanisms underlying the appreciation of innovative and renovative designs can partly explain the puzzle that renovative products are more welcomed by consumers than innovative ones. Previous studies have found that creativity not only reveals new perspectives but also promotes a sense of uncertainty that makes most people uncomfortable^12^,^13^. These feelings of uncertainty can interfere with their aptitude to appreciate creative ideas, thus, people crave creativity but always in fact reject creative ideas or products. In particular, behavioral studies have found that people may face greater uncertainty about the benefits and risks of innovative products than those of renovative products^11^,^33^, and show higher preference for renovative products compared with innovative ones^14^,^15^, partly because they feel that they lack the ability to make effective use of the radical innovations. Along these lines, the present study found that innovative designs engaged greater activation of the insula (threshold at *p* < 0.001 uncorrected), which is usually associated with negative emotion^34^,^35^,^36^. Moreover, the present study found that the construction of mental representations of renovative products evoked more activation in the motor pathway. Advanced manipulating skills representation, namely, operational imagination, might just be the critical factor needed to reduce uncertainty and the improve evaluation of renovative products. In other words, action-related experiences may provide advantages for understanding and appreciating creative designs. This finding could offer practical implications for managing innovation development, such as enriching one’s action-based experiences with new designs.

Interestingly, we found increased right PHG activation in the renovation condition compared with the innovation condition. One potential reason for this difference is that renovation may be more efficiently supported by, and thus more comprehensively integrated into, individuals’ established mental schemas. This possibility is consistent with behavioral studies on narratives, such as myths and folk tales, which are also important types of cultural innovation, in which minimally counter-intuitive narratives were better memorized and more likely to achieve cultural stability compared with radical narratives^37^. We conducted a supplementary memory experiment that was identical to the fMRI experimental procedure to determine whether renovation exhibited superior memory effects. The incidental memory performance (the free recall test) results of the 30 participants exhibited a significant difference pattern of “Renovation > Innovation > Ordinary” (see details in the Supplemental Material) and further suggested the processing priority for renovation. Both the neural and behavioral data show that renovative designs have superior memory effects. Thus, renovative designs may plausibly have better advertising and popularization effects than innovative designs.

In summary, this study demonstrated that the representation of creative designs is constructed jointly by the MTL structure and the dual pathways associated with human tools use. In particular, the mental representation of renovation and innovation may be differentially dependent on action-based experience or conceptual analysis, action-related experiences may provide advantages for appreciating new creative designs. The dissociation of cognitive brain mechanisms for processing creative designs with different invention levels not only provides a theoretical basis for understanding individual attitudes to various types of creative products but also implies approaches for launching creative designs and products.

## Methods

### Participants

Twenty undergraduate or graduate students (9 females and 11 males, aged 19–24 years old, mean age = 21.57 years, all native Chinese speakers), who were recruited from the University of Forestry Beijing or the University of Science and Technology Beijing, participated in this study as paid volunteers. All participants were right handed, had normal or corrected-to-normal vision, and did not have a history of neurological or psychiatric problems. Prior to the scanning session, the participants signed an informed consent form. The study was approved by the institutional review board of the Beijing MRI Center for Brain Research, and all methods were performed in accordance with the relevant guidelines and regulations.

### Materials

**Figure 4:**
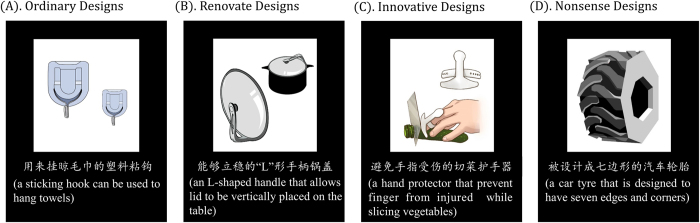
Examples of the four types of designs. Please note only the Chinese interpretations were presented in the formal fMRI experiment, the English translations of the Chinese interpretations are given here for illustration.

This study used three types of designs that varied on the level of change compared to existing things or products. The ordinary design was the typical design that individuals often encounter and use in their daily lives (Fig. 4A). In contrast, the renovative design (incrementally creative design) comprising relatively minor modifications and improvements to existing designs or products, while the primary function of the product remained unchanged. For example, an L-shaped handle lid (Fig. 4B) was modified based on a traditional round handle lid to allows the lid to be placed vertically on the table to avoid smears. The innovative design (radically creative design) exert major developments, and was created to satisfy demands that are never fulfilled by existing products. For example, a hand protector (Fig. 4C) can protect us from harm while slicing vegetables; however, individuals may not be aware of any existing products that have this function. Importantly, we try to keep a balance between renovative designs and innovative designs on the level of understandability, complexity, newness and usefulness, but a clear differences on the level of changes compared to existing things.

All designs adopted in this study included daily used items with a suitable size and an understandable functional mechanism. Thus, new products that utilized advanced technology that could be difficult for ordinary individuals to understand or large-sized products (such as large public facilities) were not used.

According to these principles, we selected 38 innovative designs, 38 renovative designs and 38 ordinary designs from a pool that contained 300 cases of creative or non-creative designs or products collected from the open resources in the internet or designed by ourselves. Additionally, we designed and arranged 23 (114 × 20%) nonsense designs (Fig. 4D) as fillers to maintain the participants’ focus on their cognitive tasks regarding the usefulness rating. These selected designs were then depicted by art professional tools to clearly demonstrate their key structures and functions in a unified, concise style. An 11- to 12-word interpretative sentence, which included the key function and name of the design, was also constructed for each design. The explanatory text may be helpful for the participants to perceive, understand, and evaluate the usefulness of the designs, particularly for the unfamiliar renovative and innovative designs.

Twenty individuals who did not participate in the formal fMRI experiment were required to rate the understandability, complexity, newness, usefulness and the level of changes compared to existing things (relative changes) of each design on a 4-point scale, which ranged from very low (1) to very high (4). The results of the rating indicated that the differences among the three conditions were significant in all five dimensions [*F*(2,58) _understandability_ = 7.95, *p* = 0.003, *η*_*p*_^2^ = 0.295; *F*(2,58) _complexity_ = 29.92, *p* = 0.000, *η*_*p*_^2^ = 0.612; *F*(2,58) _newness_ = 239.56, *p* = 0.000, *η*_*p*_^2^ = 0.927; *F*(2,58) _usefulness_ = 16.50, *p* = 0.000, *η*_*p*_^2^ = 0.465; and *F*(2,58) _relative changes_ = 450.66, *p* = 0.000, *η*_*p*_^2^ = 0.960]. Post hoc contrasts indicated that for the understandability scores, the ordinary designs were marginally higher than the renovative designs [*p* = 0.057] and significantly higher than the innovative designs [*p* = 0.000]; however, there was no difference between the renovative and innovative designs [*p* = 1.0]. For the complexity and newness, the scores for both the renovative and innovative designs were higher than those for the ordinary designs [*p*_*s*_ = 0.000]; however, there was no difference between the scores for the renovative and innovative designs [*p* = 0.250]. For the usefulness, the scores for ordinary designs were higher than those for the renovative and innovative designs [*p*_*s*_ = 0.000], whereas there was no difference between the scores for the renovative and innovative designs [*p* = 1.0] (Table 3). For the relative changes, the scores for both the renovative and innovative designs were higher than those for the ordinary designs [*p*_*s*_ = 0.000], and the scores for innovative designs higher than renovative designs [*p* = 0.000]. These behavioral data show that all dimensions of renovative and innovative designs are consistent in this study, except the level of relative changes.

### Experimental procedure

The 137 designs were randomly assigned to two runs (the first run contained 68 items; the second, 69 items). For each trial, the designs were presented together with the interpretive text for 4 sec (a pilot study found that this duration was suitable for our undergraduate participants to understand and evaluate the designs). During this stage, the participants were asked to judge whether the designs were useful or not by pressing one of the two response keys. A cross-viewing stage that jittered from 3 to 5 sec was inserted between the trials. A run lasted 9 min and 46 sec (68 trials) or 9 min and 54 sec (69 trials), and the total functional imaging time was 19 min and 40 sec. A 2-min resting interval was included between the two runs.

Immediately following the imaging session, the participants were asked to rate on a 6-point scale (from very low (1) to very high (6)) the extent to which they thought the design was new and useful. Based on the participants’ online (i.e., during the MRI scanning session) yes/no judgments regarding the designs’ usefulness (the yes/no judgment) and their offline (i.e., after the MRI scanning session) evaluations regarding the newness and usefulness (the ratings on a 6-point scale), we performed a participant-specific screening for all three types of designs evaluated by each participant. First, a design was excluded from the key analysis if the usefulness was judged online as “no” or was evaluated offline as less than 4 on the 6-point scale. Subsequently, on the dimension of newness, we excluded innovative and renovative designs that were rated less than 4, as well as ordinary designs that were rated higher than 3.

### Image acquisition

Whole-brain imaging was conducted at the Beijing MRI Center for Brain Research using a 3.0-T magnetic resonance scanner (Siemens, Erlangen, Germany) using a standard radio frequency head coil. Functional T2*-weighted images were acquired in an interleaved order using a single-shot echo-planar gradient-echo pulse sequence (TR = 2,000 ms, TE = 30 ms, FOV = 192 × 192 mm, FA = 90°, 642 matrix, 33 slices at 3.5 mm thick, and voxel size = 3.0 × 3.0 × 3.5 mm) to measure the blood oxygen level-dependent contrast. To restrict movement, the participant’s head was fixed with plastic braces and foam pads throughout the experiment. A high-resolution structural T1-weighted anatomical scan was also acquired using a three-dimensional, gradient-echo pulse sequence (TR = 2,600 ms, TE = 3.02 ms, FOV = 256 mm × 256 mm, FA = 7°, 178 slices at 1.0 mm thick, and voxel size = 1.0 × 1.0 × 1.0 mm).

### Image analysis

Imaging data were analyzed using the SPM8 software package (Statistical Parametric Mapping 8, Wellcome Department of Cognitive Neurology, Institute of Neurology, London, UK) implemented n MATLAB 2010 (MathWorks, Inc., Natick, MA, USA). For preprocessing, the images for each subject were corrected by slice-timing, realigned for head-motion correction, spatially normalized into a standard echo planar imaging (EPI) template in the Montreal Neurological Institute space, and smoothed with an 8-mm Gaussian kernel full width at half maximum (FWHM).

At the first level of analysis, three key events were defined as participant-specific valid ordinary, renovative and innovative designs. The fourth event was defined as invalid designs in all three conditions and the filled nonsense designs that were out of interest. All events were time-locked at the onset of the designs. The design matrix included one regressor for each condition, and the six movement parameters from the realignment procedure. The regressors for each condition were determined individually according to the individual responses of each participant. The “renovation & innovation > ordinary” contrast were computed and the resulting contrast images were submitted to a random-effects analysis for all participants at the second level analysis, which can help to detect brain regions associated with creative designs appreciation. For the whole-brain analyses, the threshold was generally set at *p* < 0.05, Family-Wise Error (FWE) corrected, but the threshold of *p* < 0.001 (uncorrected) was also used for detecting the activation in the PHG that was clearly expected in the study. For anatomical localization and reporting, all coordinates were transformed into Talairach space.

### Region-of-interest (ROI) analysis

In order to further detect the activation differences of creative designs appreciation related regions between the ordinary, renovative and innovative conditions exactly, a region of interest (ROI) analysis was conducted. Regions are generally appeared in the human tool use researches, and also the “renovation & innovation > ordinary” contrast were selected as ROIs. 6 anatomical areas created using WFU PickAtlas (Version3.0, http://fmri.wfubmc.edu/software/PickAtlas) were included: left IFG, PMC, SPL, IPL, and bilateral pMTG. Moreover, we also carried out a ROI analysis on the right posterior PHG, which in a prior study was reported to associate with object novelty detection and memory^38^. Right PHG ROI corresponded to the peak coordinates reported in the main effect analysis of that study, has a radius of 6 mm centered around the MNI coordinates, x = 39, y = −40, z = −14. Percent signal change in each condition was extracted for all ROIs using MarsBar toolbox (http://marsbar.sourceforge.net), and then was submitted to a one way ANOVA.

### Psychophysiological interaction (PPI) analysis

The whole-brain and ROI analyses revealed that the right PHG was more activated in both the renovative and innovative conditions than ordinary condition. Generally, the PHG integrate information from distributed neocortical regions to create or update mental representations. In order to further investigate the brain networks involved in the appreciation of renovative and innovative designs, we conducted a PPI analysis to determine whether the right PHG exhibited differential functional connectivity with other brain regions in the renovative and innovative conditions. The seed region for the connectivity analysis was a 6-mm sphere centered on the individual peak of activity in the right PHG clusters (36, −36, −14), which was identified in the “renovation & innovation > ordinary” contrasts. Two general linear models were constructed, which included one model for the neural response when understanding and evaluating the renovative designs and a second model for the innovative condition. The results for the PPI are presented at a threshold of *p* < 0.05 uncorrected, K_E_ > 10 voxels.

## Additional Information

**How to cite this article**: Huang, F. *et al*. Neural Pathway of Renovative and Innovative Products Appreciation. *Sci. Rep.* **6**, 38800; doi: 10.1038/srep38800 (2016).

**Publisher's note:** Springer Nature remains neutral with regard to jurisdictional claims in published maps and institutional affiliations.

## Supplementary Material

Supplementary Information

## Figures and Tables

**Table 1:** Brain regions associated with creative designs appreciation.

Anatomical location	L/R	BA	MNI coordinates			*t(20)*	k
			*x*	*y*	*z*		
PMC	L	6	−26	0	64	7.45	231
IFG	L	44	−54	10	24	7.33	244
IPL	L	39	−30	−74	32	6.67	115
SPL	L	7	−22	−72	60	5.45	19
pMTG	L	37	−54	−66	2	6.25	432
pMTG	R	37	58	−56	4	5.63	18
PG	L	2	−62	−30	40	6.30	148
*PHG	R	—	36	−36	−14	5.07	701

Note: Activations were significant at *p* < 0.05 FWE corrected unless otherwise specified, ^*^ means threshold was set at *p* < 0.001 uncorrected. Statistics in the *t* column show values at peak coordinates. Cluster size is represented by k. L, left; R, right. BA = Brodmann’s area. MNI = Montreal Neurological Institute. IFG, inferior frontal gyrus; IPL, inferior parietal lobule; PG, postcentral gyrus; PHG, parahippocampal gyrus; PMC, premotor cortex; pMTG, posterior middle temporal gyrus; SPL, superior parietal lobule.

**Table 2:** PPI for Renovative and Innovative designs appreciation.

Index Area	Activated Area	MNI coordinates			*t*
		*x*	*y*	*z*	
*Renovative designs* > *Innovative designs*					
Right PHG	Left PMC	−30	2	64	2.85
	Left IPL	−30	−74	40	3.44
*Innovative designs* > *Renovative designs*					
Right PHG	Left IFG	−56	6	16	4.62
	Left pMTG	−62	−56	−2	2.92

Note: Activations were significant at *p*<0.05 uncorrected. Statistics in the *t* column show values at peak coordinates. Cluster size is represented by k. MNI = Montreal Neurological Institute. IFG, inferior frontal gyrus; IPL, inferior parietal lobule; PHG, parahippocampal gyrus; PMC, premotor cortex; pMTG, posterior middle temporal gyrus.

**Table 3:** Mean (SD) scores of the behavioral assessments.

	Understandability	Complexity	Newness	Usefulness	Relative Changes
Ordinary designs	3.79 (0.29)	1.4 (0.29)	1.31 (0.39)	3.56 (0.35)	1.15 (0.14)
Renovative designs	3.46 (0.39)	2.1 (0.35)	3.12 (0.21)	3.02 (0.40)	2.48 (0.23)
Innovative designs	3.32 (0.39)	2.1 (0.31)	3.01 (0.27)	3.00 (0.36)	3.36 (0.26)
