# Supplementary material for: Neural Pathway of Renovative and Innovative Products Appreciation
Source: Sci Rep. 2016 Dec 12;6:38800. doi: 10.1038/srep38800 (PMC5150253; doi:10.1038/srep38800)
Supplement: Supplementary Information [file srep38800-s1.pdf]

Neural Pathway of Renovative and Innovative Products Appreciation

Furong Huang[1,2], Chiyue Chiu[3] & Jing Luo[1,4]

[1] Beijing Key Laboratory of Learning and Cognition, The Collaborative Innovation Center for Capital Education Development, Department of Psychology, Capital Normal University, Beijing, China
[2] College of Psychology, Jiangxi Normal University, Nanchang, China
[3] Faculty of Social Science, The Chinese University of Hong Kong, Hong Kong Special Administrative Region
[4] Key Laboratory of Mental Health, Institute of Psychology, Chinese Academy of Sciences, Beijing, China

SM1. Supplementary Memory Experiment

Participants:

Thirty undergraduate or graduate students (17 females and 13 males, aged 18–22 years old, mean age = 20.37 years, all native speakers of Chinese) recruited from Capital Normal University (Beijing, China) participated in this study as paid volunteers.

Materials:

Seventy designs (including 20 ordinary designs, 20 renovative designs, 20 innovative designs, and 10 nonsense fillers) were used in the fMRI study.

Procedure:

Seventy items of the designs were presented in a randomized order. Each item was displayed along with an interpretative text for a duration of 4 sec; during this period, the participants were asked to make a yes/no judgment on whether the designs

were useful. During the inter-stimulus interval (ISI), a fixation cross was maintained at the central location of the screen for 3-5 sec. Each picture subtended a horizontal and vertical visual angle of 9.4 °×11.4 °, and the angle of each word was 1 °×1 °, which was sufficiently clear for the participants to recognize and understand.

Following evaluation, the participants spent 5 min completing a distraction task that prevented individuals from repeating the designs. The distraction task included repetitive subtraction, beginning with a 3-digit number shown on the screen. The participants repeatedly subtracted 3 from the last number (e.g., 998, 995, 992 ……). Finally, the participants were required to make an incidental free recall on the items they had evaluated. The participants were given a sheet of blank paper and were asked to write down as many of the designs that they could in a maximum duration of 15 min.

Results:

There were 92%, 85%, and85% ordinary, renovative and innovative designs, respectively, that were evaluated by the participants as useful. For the mean reaction times (see Table1), the difference among the three conditions was significant [$F(2,58) = 73.63$, $p < .001, \eta_p^2 = .717$]. Post hoc contrasts indicated that the differences between the ordinary and renovative or innovative designs were significant [*ps* <.001]; however, there was no significant difference between the renovative and innovative designs [$p = .138$].

For the average rate of the free recall (see Table1), the difference among the three conditions was significant [$F(2,58) = 32.12$, $p < .001, \eta_p^2 = .53$]. The post hoc

contrasts indicated that the difference between any two conditions was significant [*ps* <.005], suggesting thatboth types of creative designs exhibited superior memory effects compared with the ordinary designs, whereas the renovative designs were better memorized than the innovative designs.

Table 1. Behavioral performance (Mean ± SD)

	Reaction times (ms)	Percentage of the free recall
Ordinary designs	2035 (366)	25.00 (15.41)
Renovative designs	2463 (416)	47.00 (14.42)
Innovative designs	2530 (369)	37.17 (13.52)

Note: SD represents standard deviation.
